# Advancing the predictive techno-economic and lifecycle assessment of prairie grass and manure co-digestion for renewable natural gas applications

**DOI:** 10.3389/fbioe.2025.1651510

**Published:** 2025-09-30

**Authors:** Katherine Wild, Juan Carlos Dominguez, Lisa Schulte Moore, Mark Mba Wright

**Affiliations:** ^1^ Mechanical Engineering, Iowa State University, Ames, IA, United States; ^2^ Ingeniería Química y de Materiales, Complutense University of Madrid, Madrid, Spain; ^3^ Natural Resource Ecology and Management, Iowa State University, Ames, IA, United States; ^4^ Bioeconomy Institute, Iowa State University, Ames, IA, United States

**Keywords:** ADM1, anaerobic co-digestion, renewable natural gas, techno-economic analysis, life cycle assessment

## Abstract

Prairie grass remains an underutilized agricultural resource that could provide economic, environmental, and ecological benefits to the bioeconomy. Prairie grass and manure anaerobic digestion is a promising pathway for renewable natural gas (RNG) production, but there is limited information on how co-digestion ratios impact RNG performance. This study integrates the Anaerobic Digestion Model No. 1 (ADM1) into a techno-economic analysis (TEA) and life cycle assessment (LCA) framework to evaluate RNG production via co-digestion of prairie biomass and cattle manure. Simulations across eleven feedstock ratios showed that co-digestion can increase methane yields compared to mono-digestion of prairie biomass. The highest methane production rate (227 mL/gVS) and the lowest minimum fuel selling price (MFSP) of $41.88/GJ occurred at a 1:9 prairie-to-manure volatile solids (VS) ratio. RNG yields reached 10.1 GJ/dry tonne for this configuration—39% higher than prairie-only digestion. LCA results revealed that manure-based scenarios had the lowest global warming potential (−16.0 kg CO_2_-eq/GJ), while prairie-based scenarios reduced ecotoxicity (−190 kg 2,4-D-eq/GJ). Economic and environmental benefits were further improved by accounting for biochar coproducts via system expansion and allocation. Results underscore the value of ADM1 in optimizing AD system design for both profitability and sustainability.

## Introduction

Renewable natural gas (RNG) from anaerobic digestion (AD) is becoming a widely explored energy source within the U.S. (U.S. EPA, 2023). Co-digestion, the addition of more than one substrate to a digester, is a common approach for optimizing feedstock digestibility (Hagos et al., 2017). Mono-digestion of manure can lead to process instability by supplying the reactor with a low carbon-to-nitrogen (C/N) ratio (Fernandes et al., 2009). The mono-digestion of lignocellulosic material is often infeasible due to the recalcitrant properties of the feedstock and high C/N ratios available (Karki et al., 2021; Paul and Dutta, 2018). Blending of manure and lignocellulosic material for co-digestion can provide optimal C/N ratios by balancing nutrient flows to the digester (Neshat, et al., 2017). Additional improvements in methane (CH_4_) yields from co-digestion can be attributed to an increase in microbial variations and substrate interactions within the digester (Kunatsa and Xia, 2022; Ma et al., 2020).

Several factors drive the demand for co-digestion of manure and lignocellulosic materials. Regenerative agriculture (Rhodes 2017), an alternative to single-crop farming, helps sustain food and energy production by growing feedstock that reduces soil erosion, improves nutrient retention, and provides ecological benefits. Prairie grasses provide many of these benefits (Schulte et al., 2017), but their economic value remains limited. Anaerobic digestion of lignocellulosic biomass can generate valuable fuels and chemicals (Olafasakin et al., 2024; McLaughlin et al., 2002), but this pathway faces technical and policy challenges (Groom et al., 2008; Lark 2020).

Accurate biogas modeling tools may help relieve time and economic burdens of experimentation. These models also have the potential to facilitate economic and environmental assessments of novel AD feedstocks for real-world use. Several models representing the AD process have been created, including the Anaerobic Digestion Model No. 1 (ADM1), Gaussian, and Gompertz (Emebu et al., 2022; Biernacki et al. 2013; Bułkowska et al. 2015). Modeling AD processes has become more complex throughout the years. New additions now include steps such as characterizing the feedstock by organic components, such as carbohydrates, to model the entire digestion process (Weinrich and Nelles, 2021). The International Water Association created ADM1 in 2002 (Batstone et al., 2002). ADM1 is a complex model including more than 24 dynamic variables, 19 biochemical conversion processes, and eight algebraic variables. This model encompasses five steps to represent the AD process: disintegration, hydrolysis, acidogenesis, acetogenesis, and methanogenesis.

The ADM1 model has been further expanded since 2002, incorporating various methodologies for new feedstock integration and nutrient interaction improvement (Chen et al., 2016; Koch et al., 2010; Li et al., 2020; Zhou et al., 2011). While ADM1 is widely used for biogas and CH_4_ estimation, there are still areas within the model that require further exploration, such as ammonia inhibition (Mo et al., 2023). There are few economic and environmental-focused studies utilizing ADM1 found within the literature. Glivin and Sekhar (2018) completed an economic analysis using experimental data and ADM1 to determine the cost of a digester within an institution in India. Usack et al. (2019) utilized ADM1 within an environmental assessment for an AD combined heat and power system on a dairy farm. Integrating ADM1 into economic and environmental assessments remains challenging due to the complexity of combining ADM1 with process simulation software.

This article describes a techno-economic analysis (TEA) and life cycle assessment (LCA) utilizing ADM1 for predicting the co-digestion of cattle manure and prairie biomass within BioSTEAM (Cortes-Peña, 2022; Python Software Foundation 2025). Within this study, eleven ratios of manure and prairie biomass will be analyzed, including mono-digestion and co-digestion scenarios. Our work is built upon Wild et al. (2024) for the process design of biogas upgraded to RNG. Our work also utilizes the same TEA methodology as Wild et al. (2024). An extended LCA was completed using the boundary expansion method to fully consider the impact of biochar. Additionally, we use physical, economic, and energy allocation methodologies to determine the environmental impacts of the products (RNG and biochar). To the author’s knowledge, this study demonstrates, for the first time, the integration of ADM1 and BioSTEAM, and contributes the most comprehensive predictive lifecycle assessment of prairie biomass and manure co-digestion for RNG.

## Materials and methods

### Overview

**FIGURE 1:**
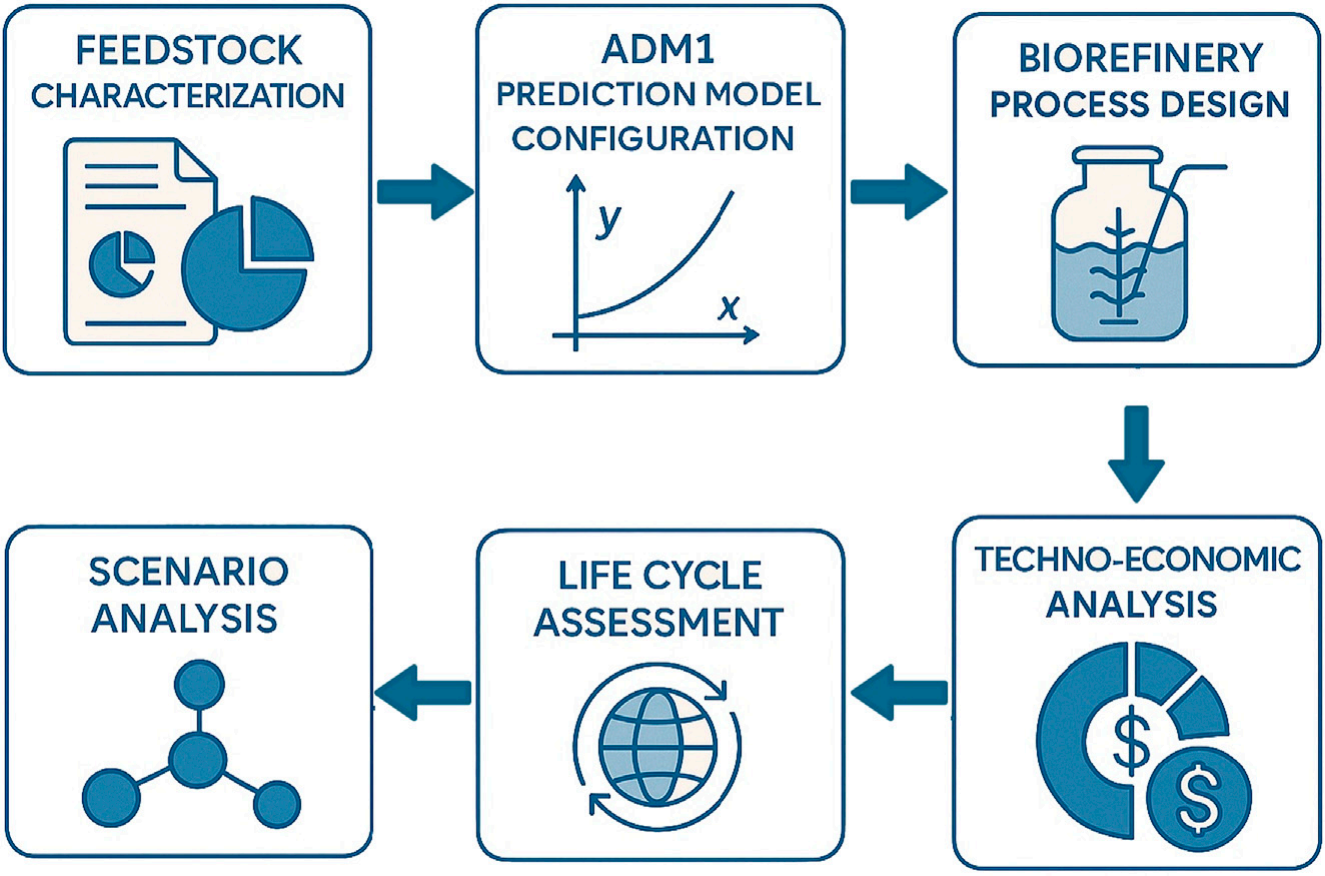
Overview of the ADM1-predictive techno-economic and lifecycle assessment of prairie biomass co-digestion for RNG production.

Figure 1 shows the methodology employed in this study. First, we gather feedstock characterization data for prairie biomass and manure. Then, we configure the ADM1 model to estimate biogas and methane production. Next, the ADM1 model is integrated with the biorefinery process design to gather facility material and energy balances. Finally, we conduct techno-economic (TEA) and lifecycle assessment (LCA) to estimate costs and environmental impacts under various co-digestion scenarios.

The working hypothesis for this study is that manure and prairie grass co-digestion offers economic and environmental benefits. Generally, these benefits vary based on the costs and environmental factors of each feedstock, and the regional context of the facility. Agricultural operations producing both manure and prairie grass, for example, could reduce their carbon footprint by increasing the contribution from the lower carbon intensity material. Synergistic effects, like increased biogas generation, are not fully explored in this study, but merit further consideration in future studies.

### Feedstock characterization

Due to the complex and comprehensive nature of the ADM1, every employment of this model should include steps for an accurate representation of the feedstocks (Klimiuk et al., 2015; Poggio et al., 2016). There are several different methodologies for substrate integration, including proper feedstock characterization, calculation of stochiometric parameters, and calibration of kinetic parameters (Koch et al., 2010; Xie et al., 2016; Zhou et al., 2011). The methodology for feedstock characterization closely follows Koch et al. (2010), where feedstocks for the model are fractioned based on the organic matter within the material. ADM1 kinetic parameters were not adjusted for this study.

**FIGURE 2:**
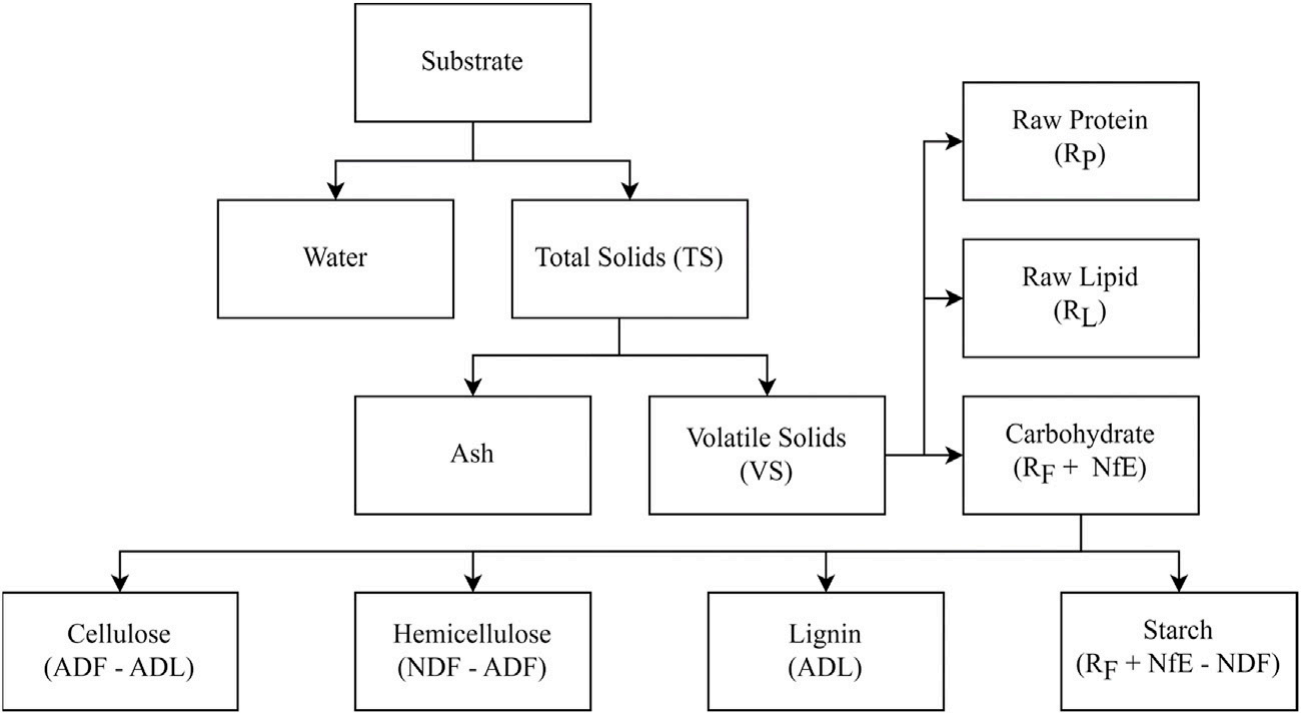
Composition breakdown of feedstocks following Koch et al. (2010).

The feedstock’s volatile solids (VS) are fractioned into proteins (R_P_), lipids (R_L_), and carbohydrates (raw fiber and nitrogen-free extract (R_F_ + NfE)). Neutral detergent fiber (NDF), acid detergent fiber (ADF), and acid detergent lignin (ADL) are used to further categorize the carbohydrate fraction as starch (R_F_ + NfE - NDF), cellulose (ADF - ADL), hemicellulose (NDF - ADF), and lignin (ADL). Figure 2 shows the composition breakdown. We obtained the composition for each feedstock from a literature review, averaging the values found for cattle manure and prairie biomass. The average of three prairie biomass species was used to categorize prairie biomass: switchgrass (*Panicum virgatum L.*), indiangrass (*Sorghastrum Nutans L.*), and big bluestem (*Andropogon Gerardii*). Table 1 summarizes the feedstocks.

**TABLE 1:** Characterization of cattle manure and prairie biomass from literature based on total solids (TS).

Fraction	Cattle manure	Switchgrass	Indiangrass	Big bluestem	Prairie biomass
Total solids (TS) (%)	17.99	91.00	91.00	91.00	91.00
Volatile solids (VS) (%TS)	84.74	93.79	92.38	91.19	92.45
Raw lipids (R_L_) (%TS)	3.26	8.39	11.01	7.86	6.78
Raw fiber (R_F_) (%TS)	26.59	49.6	44.24	33.19	42.34
Raw proteins (R_P_) (%TS)	13.74	5.89	8.14	7.53	7.19
NDF (%TS)	47.73	71.30	69.23	69.10	69.88
ADF (%TS)	36.54	43.06	37.97	36.39	39.14
ADL (%TS)	14.96	5.35	3.8	3.80	4.32
References	Gaballah et al. (2020), Jabłoński et al. (2015), Liao et al. (2007), Lucas et al. (1975), Müller (1980)	Backus (2014), Butkutė et al. (2013), Dien et al. (2006), Kieffer et al. (2023), Pearson et al. (1982), Seepaul et al. (2016)	Backus (2014), Moore and Buxton (2000); Ogunlade (2012), Oloyede (2013), USDA (2008), Waller et al. (1972)	Moore and Buxton (2000), Ogunlade (2012), Oloyede (2013), Pearson et al. (1982)	This study^a^

^a^ The “Prairie Biomass” represents the average of the three biomass species. All prairie biomass is dried at the same rate, following Khanchi and Birrell (2017).

After substrate characterization, NfE was calculated following Zhou et al. (2011), shown in Equation 1.

$$NfE+R_{F}=VS-R_{P}-R_{L}$$ (1)

### Integration of ADM1 for manure and prairie grass Co-digestion modelling

As ADM1 quantifies the system with respect to the chemical oxygen demand (COD), the theoretical oxygen demand (ThOD) is used to quantify the degradability of proteins, lipids, starch, cellulose, hemicellulose, and lignin. As lignin has no defined structure, Nadji et al. (2009) determined the formula from *Stipa Tenacissima (L.).* Lignin is entirely inert within this model, while lipids, proteins, and starches are entirely biodegradable. Cellulose and hemicellulose are partially degradable. Table 2 displays the ThOD for each fraction within the model (Koch et al., 2010).

**TABLE 2:** Theoretical Oxygen Demand (ThOD) of substrate fractions.

Substrate	Elemental formula	ThOD (g_O2_/g_TS_)
Protein (ThOD_P_)	C_5_H_7_O_2_N	1.42
Lipid (ThOD_L_)	C_57_H_104_O_6_	2.90
Starch, cellulose, hemicellulose (ThOD_Ch_)	(C_6_H_10_O_5_)_n_	1.19
Lignin (ThOD_I_)	C_10.92_H1_14.24_O_5.76_	1.56

The composite material (X_c_) for each feedstock was calculated via the summation of the ThOD for each fraction multiplied by the amount of the fraction present from the feedstock, shown in Equation 2 (expressed in kgCOD/m^3^). The density (ρ) of each substrate inside the digester was assumed to be 1,000 kg/m^3^.

$$X_{C}=\left(\rho \right)\left(TS\right)\left[R_{P}\left({ThOD}_{P}\right)+R_{L}\left({ThOD}_{L}\right)+ADL\left({ThOD}_{I}\right)+\left(R_{F}+NfE-ADL\right)T{hOD}_{Ch}\right]$$ (2)

The degradation percentages (D_VS_) for cattle manure and prairie biomass were assumed to be 49.5% and 36.1%, respectively (Jabłoński et al., 2015; Niu et al., 2015). We also integrated fixed stoichiometric parameters, f-factors, from Koch et al. (2010) for the feedstock characterization. These f-factors (f_Ch_xc_, f_L_xc_, f_P_xc_, and f_I_xc_) allocate the various component fractions of X_C_, following the disintegration step as carbohydrates (X_Ch_), lipids (X_L_), proteins (X_P_), and inert material (X_I_). Utilizing f-factors ensures the sum of all components equate to X_C._ Calculations for the f-factors utilize a degradation coefficient, d (%), calculated from the determined degradability of the substrate (D_VS_) and the substrate composition. The degradation coefficient, shown in Equation 3, determines the degradable parts of cellulose and hemicellulose. Equations 4–7 show the f-factor calculations, all represented in terms of kgCOD/kgCOD.

$$d=\frac{NDF-VS\left(1-D_{VS}\right)}{NDF-ADL}$$ (3)

$$f_{Ch\_xc}=\frac{\left(R_{F}+NfE-NDF\right)+\left(NF-ADL\right)d}{VS}$$ (4)

$$f_{L\_xc}=\frac{R_{L}}{VS}$$ (5)

$$f_{P\_xc}=\frac{R_{P}}{VS}$$ (6)

$$f_{I\_xc}=\frac{ADL+\left(NDF-ADL\right)\left(1-d\right)}{VS}$$ (7)

Additional characterization is required for manure, as the substrate has already been partially disintegrated from the cow’s rumen (Zhou et al., 2011). Soluble components for monosaccharides (S_su_), amino acids (S_aa_), long-chain fatty acids (S_fa_), and inerts (S_i_) are calculated based on the X_c_, then subtracted from the individual components as Zhou et al. (2011) proposed. S_su_ was subtracted from X_ch_, S_aa_ was subtracted from X_pr_, S_fa_ was subtracted from X_li_, and S_i_ was subtracted from X_i._ Table 3 highlights the soluble percentages within manure from Wichern et al. (2007). As acetate’s solubility was determined to be 0.0 by Wichern et al. (2009), it was not considered in calculations.

**TABLE 3:** Soluble components from disintegration of cattle manure.

Parameter	Indicator	Solubility (% COD)
S_su_	Monosaccharides	10.8
S_aa_	Amino acids	2.7
S_fa_	Total long chain acids	0.8
S_i_	Inerts	5.7

Finally, each feedstock was calibrated using trial and error based on the ADM1 calibration parameters available within the literature for similar feedstocks (Lübken et al. 2007; Shi et al. 2014; Thamsiriroj and Murphy 2011). Supplementary Tables S1, S2 include information on the ADM1 calibration parameters. Various calibration parameters were tested. Parameters were chosen based on how closely the biogas and CH_4_ model results matched experimental CH_4_ production rates found within the literature. All other parameters are determined by the benchmark simulation model no. 2 (Rosén and Jeppsson, 2006). As the model is dependent on the feedstock characterization shown in Table 1, new feedstock parameters could widely impact the predicted results of the model. Additionally, there was little literature available for co-digestion calibration parameters at different ratios. All co-digestion scenarios utilized the same calibration parameters, so the amount of each co-digested feedstock had no effect on the calibration parameters chosen. Previous studies have indicated that these are some of the limitations of ADM1, and new methods have been proposed to handle dynamic feedstock properties (Long et al., 2025).

### Biorefinery process design

**FIGURE 3:**
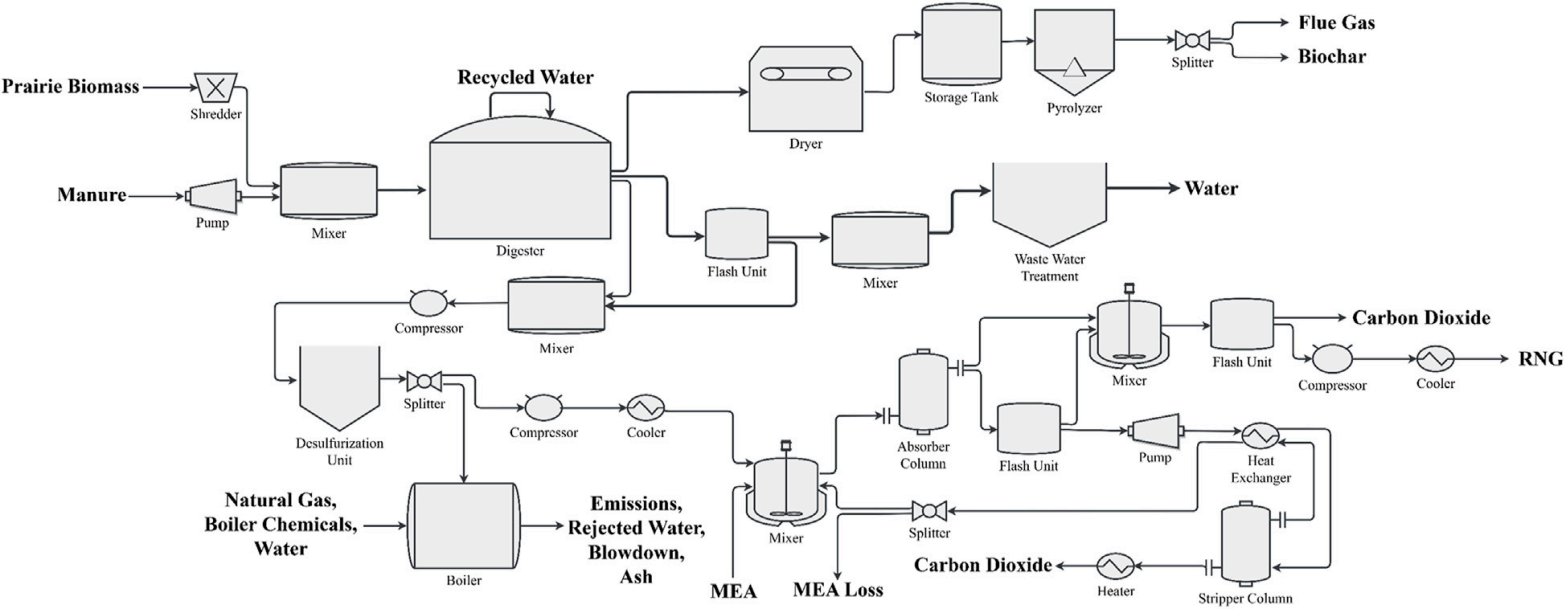
Full process diagram of a commercial biorefinery for the co-digestion of prairie biomass and cattle manure.

We utilized version 2.37.4 of BioSTEAM in Python 3.11 (Cortes-Peña, 2022) for the system design and implementation of the ADM1. Wild et al. (2024) was used as a basis for biogas upgrading to RNG from the co-digestion of prairie biomass and cattle manure. While Wild et al. (2024) includes a pretreatment for biomass, we chose to simplify the inputs to the ADM1 model to focus on feedstock characterization, without the pretreatment process. The feedstocks are digested with a hydraulic retention time of 30 days under mesophilic conditions. Longer hydraulic retention times would increase biogas yields resulting in lower per-unit costs and environmental impacts. However, increasing the hydraulic retention time has diminishing returns after a certain duration, and capital cost increases can overtake cost reductions. The digestate is thermally treated for eventual soil application. After biogas is collected, hydrogen sulfide is removed from the gas stream. On-site combustion is completed to meet electricity and heat requirements, burning 5% of the biogas stream and purchased natural gas as needed. Carbon dioxide (CO_2_) is removed from the gas stream by an amine scrubber system with monoethanolamine (MEA). The full process diagram is shown in Figure 3.

There were 11 ratios examined, each scenario changing based on the VS ratio of prairie biomass (B) and cattle manure (M). The rate of material entering the digester is 1,000 kgVS/hr for all scenarios. Scenario B10:M0 requires 100% of the VS makeup from biomass, while B9:M1 requires 90% of the VS makeup from biomass and 10% from manure, and so on. The digester’s recycled water makes up 10% TS within the reactor for each scenario. These ratios were selected to balance the tradeoffs between not having enough scenarios to capture second-order effects, like feedstock synergies (Rahic et al., 2024; Wani and Parveez 2025), and generating too many scenarios for an accessible analysis of the trends.

### Techno-economic analysis

The capital costs of the BioSTEAM process units have been modified following Wild et al. (2024), including cost estimates for the digester system, biomass shredder, and gas compressor. Wild et al. (2024) determined several operating parameters to be sensitive for the estimation of the minimum fuel selling price (MFSP) of RNG. These parameters include the cost of the prairie biomass, cattle manure, biochar, and the internal rate of return (IRR). For further analysis, we are expanding upon those four parameters via an additional literature review. Wild et al. (2024) displays the full sensitivity and uncertainty analyses completed for this system.

Wild et al. (2024) determined the cost of prairie biomass from the break-even farm gate prices for switchgrass, at $105/tonne. Krohn (2015) reported that farmers selling switchgrass for $120/tonne are profitable and may have the potential to outcompete corn and soy on low-quality land. Additionally, the price of manure is now based on application rates of liquid manure to a crop field, calculated to be $41/tonne (Olsen and Leibold, 2022). While biochar prices in literature range extensively, Thengane et al. (2021) estimated the product to have a minimum selling price of $150/tonne. Finally, Rajendran et al. (2019) utilized an IRR of 7% for a biogas upgrading plant receiving incentives. All streams with associated costs are shown in Table 4.

**TABLE 4:** Prices for all feedstocks, chemicals, or co-products within the system.

Streams	Price ($/tonne)
Cattle manure	41
Prairie biomass	120
Natural gas	245
MEA	1,080
Biochar	150

### Life cycle assessment

Following the ISO 14,040 and 14,044 LCA framework, the goal of this attributional LCA is to compare impacts from various co-digestion ratios of cattle manure and prairie biomass (ISO, 2006a; ISO, 2006b). The system boundary expansion method is used to quantify effects due to the biochar, including the substitution of nitrogen (N) synthetic fertilizer, as biochar has several beneficial nutrient qualities (Ding et al., 2016; Wang et al., 2022). Additionally, allocation methods are used to determine the responsibility of environmental impact for each product (RNG and biochar) within the mono-digestion scenarios. Mass, energy, and economic allocation methods are used to expand upon the original LCA completed in Wild et al. (2024) to fully understand the impact of allocation methodologies. The boundary of our LCA is well-to-gate, where any physical streams crossing that boundary will be quantified as either feedstock, waste, or product.

We used EcoInvent V3.7 within OpenLCA V2.1 as our environmental impact factor database (Ecoinvent, 2025; OpenLCA, 2025). The Tool of the Reduction and Assessment of Chemical and other environmental Impacts (TRACI) was used for quantification of all streams flowing between the defined scope boundaries (Bare, 2011). TRACI was developed by the U.S. Environmental Protection Agency for conducting LCAs. We analyzed nine different impact categories within this system: one climate change impact category (global warming), three ecosystem quality impact categories (eutrophication, acidification, and ecotoxicity), three categories impacting human health (respiratory effects, carcinogenics, and non-carcinogenics), and two categories impacting both human health and ecosystem quality (ozone layer depletion and photochemical oxidation). The functional unit was considered as 1 GJ of RNG.

A transportation distance of 50 km was considered for chemicals entering the plant. Transportation was not considered for cattle manure and biomass, as they were both assumed to be located near the facility. Natural gas is transported to the facility within a pressurized pipeline. The biomass entering the plant was grass produced on a permanent grassland, therefore no additional land-use change impacts were considered beyond the feedstock-specific factor. We consider cattle manure to be an avoided waste. Synthetic N fertilizer was avoided on a 1:1 mass basis (Han et al., 2013). The total N content of biochar derived from cattle manure digestion is 1.71% of the total dry wt% (Schouten et al., 2012). The total N content of biochar derived from the digestion of herbaceous biomass and agro-industrial residues is 1.78% of the total dry wt% (Miliotti et al., 2020). Biochar N levels were determined from the feedstock ratios for each scenario. A system-wide CH_4_ leak was considered for the plant at a 2% volume basis of RNG collected, to consider the real-world impacts of a typical gas biorefinery. The LCA inventory can be seen in Table 5.

**TABLE 5:** LCA impact factor inventory from EcoInvent for the boundary expansion method for inclusion of synthetic N fertilizer offset.

Impact category potential	Prairie biomass	Cattle manure	Avoidance of synthetic N fertilizer	MEA	Natural gas	CH_4_ leak
Acidification (mol H+ eq./kg)	1.88 × 10^−2^	−6.16 × 10^−3^	−1.83	4.7 × 10^−1^	9.25 × 10^−2^	0
Ecotoxicity (kg 2,4-D eq./kg)	−1.04	−1.89 × 10^−2^	−4.56	1.05	8.99 × 10^−2^	2.45 × 10^−7^
Eutrophication (kg N/kg)	3.09 × 10^−4^	−1.93 × 10^−5^	−6.29 × 10^−3^	1.03 × 10^−2^	7.05 × 10^−5^	0
Global Warming (kg CO_2_ eq./kg)	5.60 × 10^−2^	−3.15 × 10^−2^	−9.7	3.36	4.87 × 10^−1^	1.35 × 10^1^
Ozone Depletion (kg CFC-11 eq./kg)	5.80 × 10^−9^	−1.80 × 10^−9^	−4.88 × 10^−7^	1.43 × 10^−7^	2.38 × 10^−7^	0
Photochemical Oxidation (NO_x_ eq./kg)	3.68 × 10^−4^	−6.24 × 10^−5^	−1.78 × 10^−2^	5.41 × 10^−3^	8.54 × 10^−4^	1.73 × 10^−3^
Carcinogenics (kg benzene eq./kg)	−6.69 × 10^−5^	−4.96 × 10^−5^	−1.09 × 10^−2^	4.32 × 10^−2^	8.32 × 10^−4^	0
Non-Carcinogenics (kg toluene eq./kg)	−1.30 × 10	−2.55 × 10^−1^	−5.55 × 10	1.09 × 10	4.54	1.50 × 10^−3^
Respiratory Effects (kg PM_2.5_ eq./kg)	9.08 × 10^−5^	−2.90 × 10^−5^	−7.73 × 10^−3^	3.27 × 10^−3^	3.47 × 10^−4^	0

Biochar offers several agricultural and environmental benefits to AD and their ecosystems (Archontoulis et al., 2016; Laird et al., 2009; Uddin et al., 2022). Previous studies have shown that adding biochar to the digester can increase biogas yields and quality leading to lower RNG prices and carbon footprints (Uddin et al., 2022). Furthermore, biochar serves as a nutrient recycling and carbon sequestration agent. Finally, biochar could have long-term benefits such as crop yield and soil organic carbon content increases that are topics of active investigation.

The lifecycle impacts of prairie grass cultivation on land use and fertilizer dynamics is a research area of growing interest (Li et al., 2025). Several groups are collecting the field data to improve the modeling of prairie grass landscapes (Edmonds et al., 2021). These factors could significantly impact the environmental performance of grass AD system. This study relies on factors available in EcoInvent, which may require updates to reflect modern practices. A detailed characterization of the land use and fertilizer displacement impact factors is outside the scope of the present study.

An additional analysis was completed for the allocation of emissions based on the mass, economic, and energy content of the system products: RNG and biochar. The total impacts of each mono-digestion scenario were allocated based on the metric used and the production rates of the products. The mass allocation metric is based on the total mass production rate. The economic allocation metric is based on the estimated price (MFSP) for biochar and RNG. The energy content of biochar and RNG was determined to be 22 MJ/kg and 52.5 MJ/kg, respectively (Lee et al., 2020).

Sensitivity analysis of key model parameters was conducted to evaluate their impacts on the MFSP and the GWP. MFSP sensitivity parameters include RNG yield, manure, biomass, biochar, and natural gas prices, and IRR. GWP sensitivity parameters include manure and biomass flow rate and emission factor, RNG yield, nitrogen fertilizer displacement, and emission factors for CH_4_ leakage and natural gas (purchased and avoided). Each of these parameters was varied ±20% to capture the proportional impact on the output variable.

## Results

### Simulated system results

The inlet concentration, X_c_, was calculated for each feedstock before digestion, equaling 166 kgCOD/m^3^ and 1,136 kgCOD/m^3^ for manure and prairie biomass, respectively. The degradation coefficient, d, used for determining the degradability of cellulose and hemicellulose, was larger for all co-digestion scenarios when compared to the mono-digestion scenarios. Additionally, f-factors f_Ch_xc_ and f_P_xc_ linearly increased with the addition of manure, while f_I_xc_ and f_L_xc_ linearly increased with the addition of biomass. Additional calculated parameters for the model are presented in the Supplementary Material.

**FIGURE 4:**
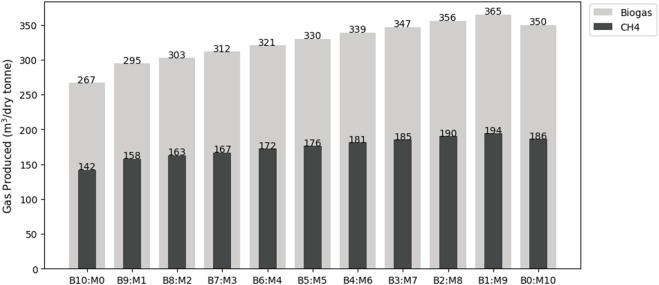
Biogas and CH_4_ output predictions from the digestion of prairie biomass and cattle manure.

The mono-digestion of prairie biomass produced 156 mL/gVS of CH_4_ while the mono-digestion of manure produced 219 mL/gVS of CH_4_. All co-digestion scenarios had larger CH_4_ production rates than the mono-digestion of prairie biomass. Two out of the nine co-digestion scenarios produced larger CH_4_ rates than the mono-digestion of manure (B8:M2 and B9:M1), with the highest CH_4_ production rate at 227 mL/gVS (B9:M1). The production of CH_4_ and biogas in shown in Figure 4.

As the model is based on the VS content of the feedstocks, manure is required in a much greater quantity than biomass within our system. As a result, the two mono-digestion scenarios have a flow rate of 6,560 kg/hr and 1,210 kg/hr for manure and biomass, respectively. Digestate production levels increase with the addition of biomass, with the largest production rate of 557 kg/dry tonne (B10:M0) due to the recalcitrant nature of the biomass. B1:M9 has the largest CH_4_ production from biogas, while B0:M10 has the largest residual CH_4_ collected from the digestate. The residual CH_4_ from the digestate increases linearly with the addition of manure, from 57 m^3^/dry tonne to 139 m^3^/dry tonne. B1:M9 is the largest producer of RNG at 10.1 GJ/dry tonne. The RNG produced in B1:M9 is 39% larger than the RNG produced in B10:M0. Further results are displayed in Table 6.

**TABLE 6:** Production rates for RNG and biochar for all scenarios.

Scenario	Biochar production rate (kg/dry tonne)	RNG production rate (GJ/dry tonne)
B10:M0	557	6.2
B9:M1	545	7.0
B8:M2	533	7.4
B7:M3	521	7.7
B6:M4	509	8.1
B5:M5	497	8.5
B4:M6	485	8.9
B3:M7	47	9.3
B2:M8	460	9.7
B1:M9	448	10.1
B0:M10	436	10.1

### Techno-economic analysis

**FIGURE 5:**
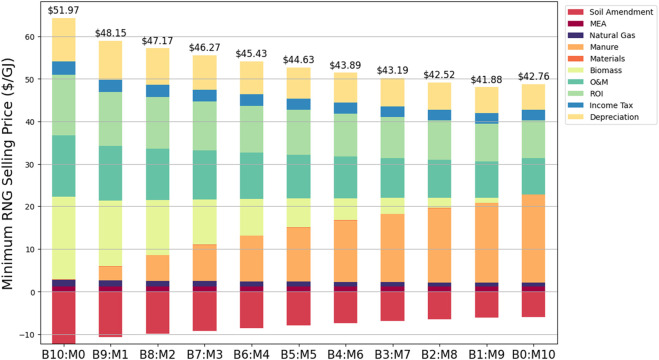
Minimum renewable natural gas (RNG) fuel selling price for prairie grass (B) and manure (M) ratios ranging from B10:M0 to B0:M10.

The estimated MFSP for all scenarios ranged from $41.88/GJ to $51.97/GJ, as shown in Figure 5. The MFSP is heavily dependent on the production of CH_4_ from digestion, as B1:M9 has the smallest estimated MFSP from an increase in feedstock efficiency and residual CH_4_ from the digestate. The difference in feedstock pricing does not have a large effect on the MFSP. This is due to the VS content difference, as the biomass has more digestible material on a mass basis. This results in a slightly larger cost for manure acquisition, as the total feedstock costs for B10:M0 and B0:M10 are $19.38/GJ and $20.60/GJ, respectively.

The MFSP offset from the biochar increases with the addition of biomass and larger N content found within the biochar. This results in an offset of $5.91/GJ and $12.27/GJ for scenarios B0:M10 and B10:M0, respectively. Operating and maintenance (O&M) costs make up between 20% and 28% of the total MFSP. Return on investment (ROI) costs range from 21% to 27%. As the amine used for CO_2_ removal (MEA) is recycled, it does not have a large effect on the MFSP ($1.21/GJ to $1.25/GJ). These costs are comparable to those reported in previous studies, which range from $10 to $100/GJ (Bhatt and Tao 2020).

Capital costs within the system ranged from $13.31 million (B10:M0) to $13.84 million (B1:M9), with significant costs coming from the stripper column, boiler, and digester. This relationship closely mirrors the biogas production rates, as more biogas and CH_4_ collected require larger downstream units for upgrading. For example, the absorber column equipment cost increased by 37% for B1:M9 compared to B10:M0. All scenarios within this plant have a net present value (NPV) to reach a breakeven point. B0:M10 had the largest annual variable operating cost due to a larger amount of feedstock required to meet VS feed conditions. B1:M9 had the largest calculated value of annual operating costs subtracted from the yearly sales of products.

These costs are representative of small-to medium-scale anaerobic digestion systems. Costs for larger systems may vary due to the benefits of economies-of-scale and challenges of increased logistical and complexity costs (Wright and Brown 2007). We believe most of the conclusions regarding varying feedstock type ratios will remain relevant at any scale, but this will require validation at relevant commercial scales.

### Life cycle assessment

**FIGURE 6:**
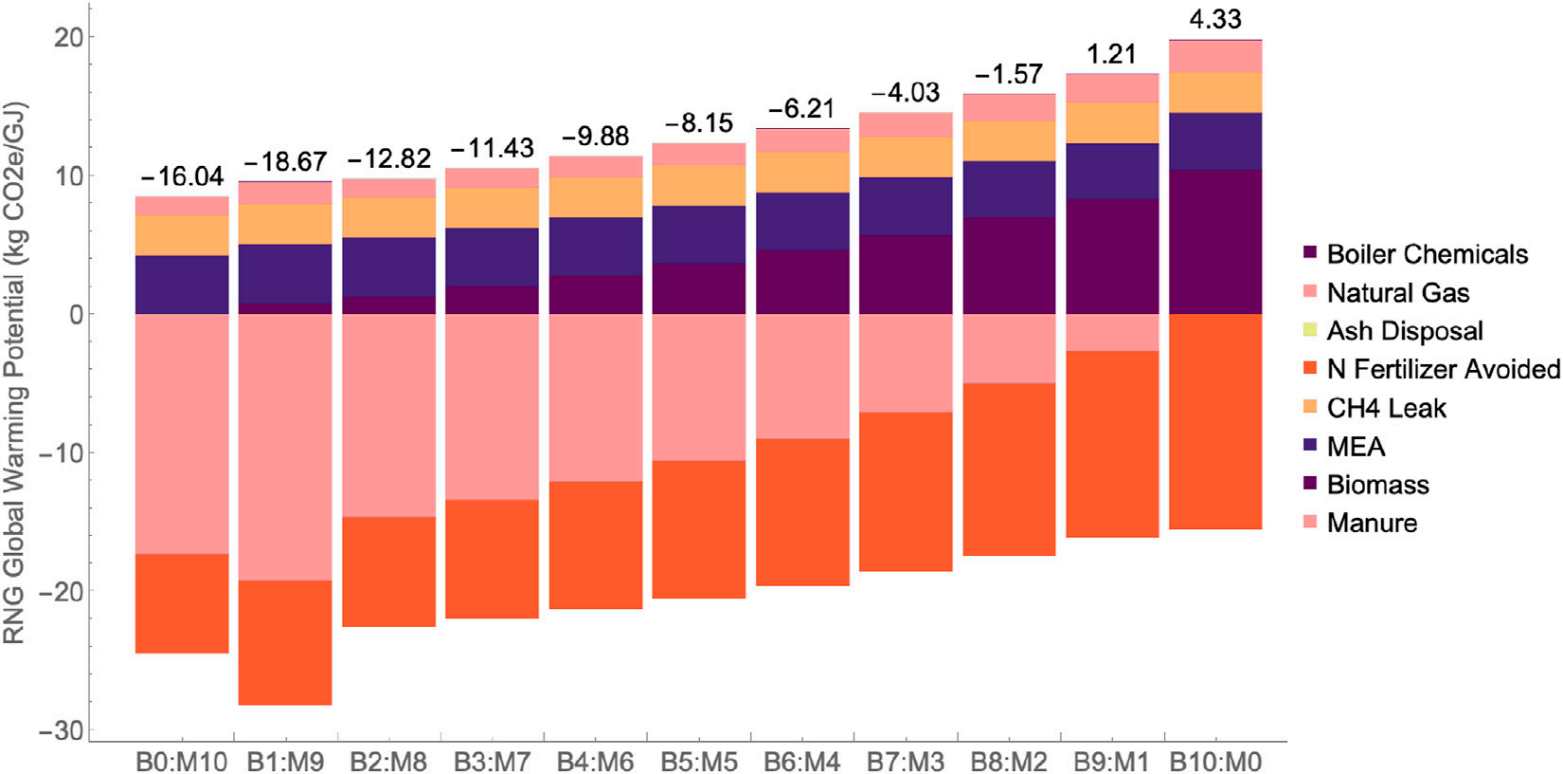
Renewable Natural Gas (RNG) Global Warming Potential (GWP) for prairie grass (B) and manure (M) ratios ranging from B10:M0 to B0:M10.

The LCA boundary of our AD upgrading facility was expanded to include impacts of biochar for the avoidance of synthetic N fertilizer. The total impact of the system is largely dependent on what feedstocks are being utilized, as manure has avoided burdens in all categories due to being an avoided waste. All avoided burdens are represented with negative values, while burdens impacting the system are represented with positive values. The GWP from the feedstocks alone ranged from −16.0 kg CO_2_ eq./GJ to 4.33 kg CO_2_ eq./GJ for B0:M10 and B10:M0, respectively. Figure 6 shows how the GWP varies for different feedstock ratios These values are comparable to commercial carbon intensity (CI) scores reported in the California Low Carbon Fuel Standard (LCFS) database for anaerobic digestion. LCFS CI scores for RNG range from −680 to 110 kg CO_2_ eq./GJ, with lignocellulosic biomass pathways ranging between −20 and 20 kg CO_2_ eq./GJ (California Air Resource Board (CARB), n. d.).

**FIGURE 7:**
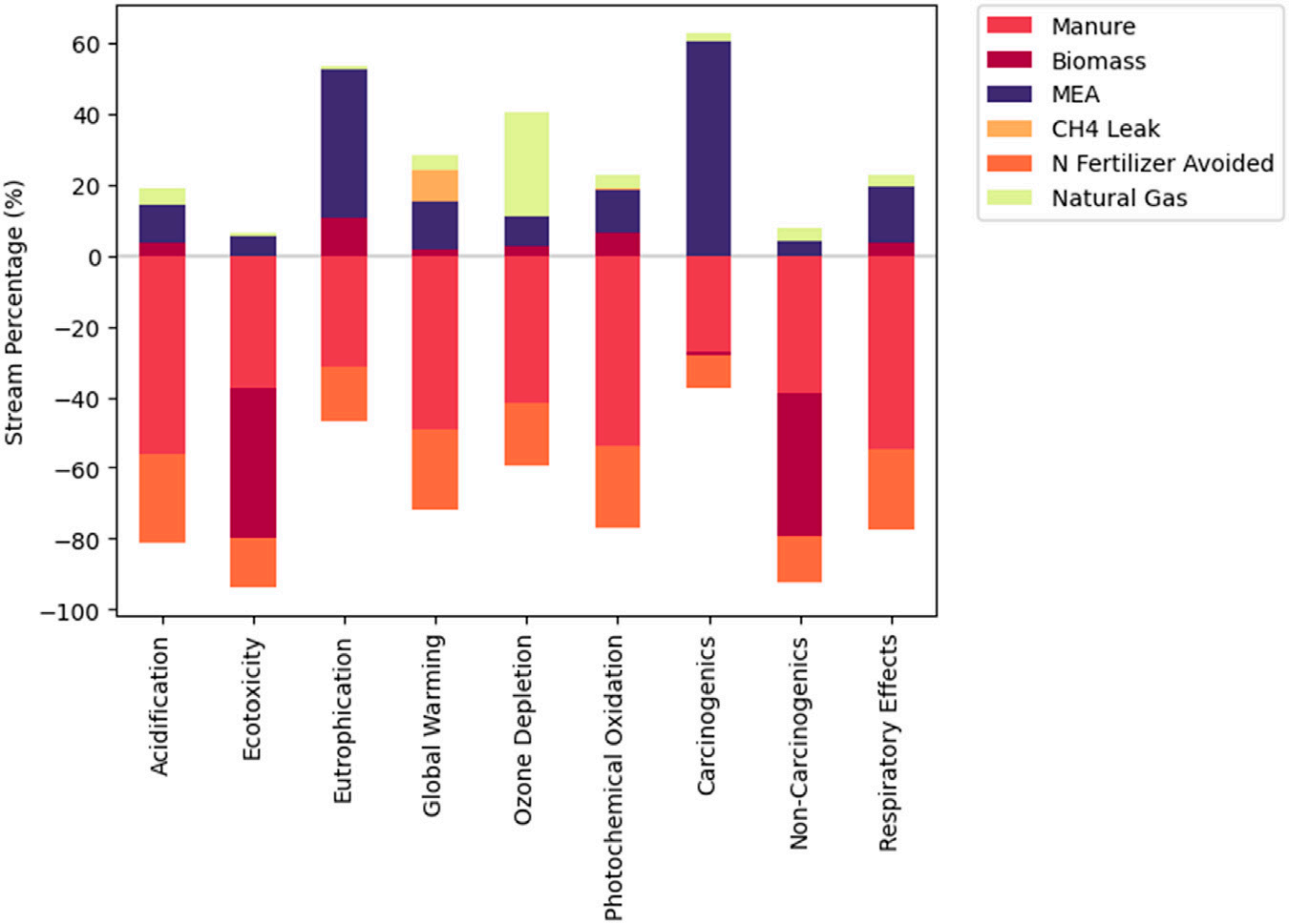
Percentage breakdown of LCA results with each category summing to 100% for B1:M9.

Biomass integration had a large estimated avoided burden for the ecotoxicity potential, with the estimated net ecotoxicity ranging from −190 kg 2,4-D eq./GJ for B10:M0 (as compared with −12 2,4-D eq./GJ for B0:M10). The net non-carcinogenic potential is also lower for B10:M0 at −2,350 kg toluene eq./GJ when compared to B0:M10 at −160 kg toluene eq./GJ. Figure 7 shows the results of the nine impact categories for the scenario with the smallest estimated MFSP (B1:M9).

The CH_4_ leak within the system did not vary significantly, as the global warming potential for all scenarios was between 2.91 kg CO_2_ eq./GJ and 2.92 kg CO_2_ eq./GJ. The largest contributors to the eutrophication potential are biomass cultivation (B4:M6 to B10:M0) and MEA use (B0:M10 to B3:M7). MEA is also the largest contributor to the carcinogenic potential for all scenarios. Natural gas was the largest burdening factor to ozone depletion potential, with the largest contribution at 1.14 × 10^−6^ kg CFC-11 eq./GJ for B10:M0. Biochar had a significant influence on all categories, due to the avoidance of synthetic N fertilizer. Further results can be seen in Table 7.

**TABLE 7:** Burdens, gains, and net values for each impact category are displayed for B10:M0, B1:M9, and B0:M10.

Indicator breakdown	B10:M0	B1:M9	B0:M10
Net Gain: Acidification Potential (mol H+ eq./GJ)	4.37	1.03	8.42 × 10^−1^
Net Loss: Acidification Potential (mol H+ eq./GJ)	−2.93	−4.47	−4.75
Net Acidification Potential (mol H+ eq./GJ)	**1.44**	−**3.44**	−**3.91**
Net Gain: Eutrophication Potential (kg N/GJ)	6.78 × 10^−2^	1.64 × 10^−2^	1.33 × 10^−2^
Net Loss: Eutrophication Potential (kg N/GJ)	−1.00 × 10^−2^	−1.44 × 10^−2^	−1.53 × 10^−2^
Net Eutrophication Potential (kg N/GJ)	**5.77** × **10** ^**−2**^	**1.94** × **10** ^**−3**^	−1**.97** × **10** ^**−3**^
Net Gain: Ecotoxicity Potential (kg 2,4-D eq./GJ)	1.75	1.57	1.59
Net Loss: Ecotoxicity Potential (kg 2,4-D eq./GJ)	−1.92 × 10^2^	−2.36 × 10	−1.38 × 10
Net Ecotoxicity Potential (kg 2,4-D eq./GJ)	**−1.90** × **10** ^**2**^	**−2.20** × **10**	**−1.22** × **10**
Net Gain: Global Warming (kg CO_2_ eq./GJ)	1.93 × 10	9.00	8.46
Net Loss: Global Warming Potential (kg CO_2_ eq./GJ)	−1.55 × 10	−2.31 × 10	−2.45 × 10
Net Global Warming Potential (kg CO_2_ eq./GJ)	**4.33**	−**1.41** × **10**	−**1.60** × **10**
Net Gain: Ozone Depletion Potential (kg CFC-11 eq./GJ)	2.37 × 10^−6^	8.78 × 10^−7^	8.16 × 10^−7^
Net Loss: Ozone Depletion Potential (kg CFC-11 eq./GJ)	−7.79 × 10^−7^	−1.27 × 10^−6^	−1.35 × 10^−6^
Net Ozone Depletion Potential (kg CFC-11 eq./GJ)	**1.59** × **10** ^**−6**^	−**3.93** × **10** ^**−7**^	−**5.37** × **10** ^**−7**^
Net Gain: Photochemical Oxidation Potential (kg NOx eq./GJ)	7.64 × 10^−2^	1.32 × 10^−2^	9.51 × 10^−3^
Net Loss: Photochemical Oxidation Potential (kg NOx eq./GJ)	−2.84 × 10^−2^	−4.46 × 10^−2^	−4.75 × 10^−2^
Net Photochemical Oxidation Potential (kg NOx eq./GJ)	**4.80** × **10** ^**−2**^	−**3.14** × **10** ^**−2**^	−**3.80** × **10** ^**−2**^
Net Gain: Respiratory Effects Potential (kg PM_2.5_ eq./GJ)	2.18 × 10^−2^	5.98 × 10^−3^	5.09 × 10^−3^
Net Loss: Respiratory Effects Potential (kg PM_2.5_ eq./GJ)	1.24 × 10^−2^	−2.03 × 10^−2^	−2.17 × 10^−2^
Net Respiratory Effects Potential (kg PM_2.5_ eq./GJ)	**9.46** × **10** ^**−3**^	−**1.43** × **10** ^**−2**^	−**1.66** × **10** ^**−2**^
Net Gain: Carcinogenic Potential (kg benzene eq./GJ)	5.71 × 10^−2^	5.65 × 10^−2^	5.71 × 10^−2^
Net Loss: Carcinogenic Potential (kg benzene eq./GJ)	−2.92 × 10^−2^	−3.37 × 10^−2^	−3.54 × 10^−2^
Net Carcinogenic Potential (kg benzene eq./GJ)	**2.79** × **10** ^**−2**^	**2.28** × **10** ^**−2**^	**2.17** × **10** ^**−2**^
Net Gain: Non-Carcinogenic Potential (kg toluene eq./GJ)	3.56 × 10	2.58 × 10	2.59 × 10
Net Loss: Non-Carcinogenic Potential (kg toluene eq./GJ)	−2.39 × 10^3^	−3.02 × 10^2^	−1.82 × 10^2^
Net Non-Carcinogenic Potential (kg toluene eq./GJ)	**−2.35** × **10** ^**3**^	**−2.77** × **10** ^**2**^	**−1.56** × **10** ^**2**^

We also considered an attributional LCA based on the mass, energy, and economics of each product. Table 8 displays the percentage breakdown used for assigning impacts to RNG and biochar for both mono-digestion scenarios to see the range of possible outcomes. Biochar received the majority of impacts for mass allocation due to the product’s physical properties. Energy allocation favors the RNG due to its higher energy content. Economic allocation places most of the impact on RNG due to the significant price difference of the products. Scenarios with a larger use of manure place slightly more of an impact on the RNG for all allocation methods, as there is a greater production of RNG (seen through comparing B10:M0 and B0:M10).

**TABLE 8:** Percentage allocation of environmental and human impact categories for biochar and RNG.

Scenario	B10:M0	B0:M10
Mass Allocation: Biochar	82.49%	69.39%
Mass Allocation: RNG	17.51%	30.61%
Energy Allocation: Biochar	66.37%	48.72%
Energy Allocation: RNG	33.62%	51.28%
Economic Allocation: Biochar	20.57%	13.15%
Economic Allocation: RNG	79.43%	86.85%

The total global warming potential for B10:M0 was estimated at 19.32 kg CO_2_ eq./GJ without considering the impacts of biochar. Utilizing the above allocation emissions, RNG is responsible for 3.38 kg CO_2_ eq./GJ (mass), 6.50 kg CO_2_ eq./GJ (energy), or 15.35 kg CO_2_ eq./GJ (economic), depending on the allocation method used. A more detailed discussion on the impact of various allocation methods on biochar and RNG systems is discussed in the study by Wild et al. (2024).

### Sensitivity analysis

**FIGURE 8:**
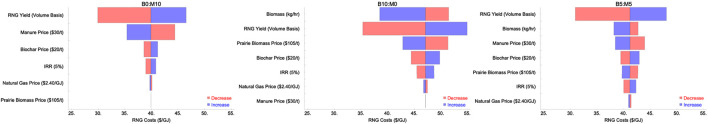
Sensitivity analysis of Renewable Natural Gas (RNG) costs to ±20 changes in RNG yield, material prices, and Internal Rate of Return (IRR).

The results of the MFSP sensitivity analysis are shown in Figure 8. RNG yield is the most impactful parameters under most scenarios followed by the feedstock and biochar prices. The IRR has a lower relative impact. Twenty percent changes in these parameters vary the RNG costs by up to $20/GJ. This suggests that there is significant value in accurately predicting AD performance to minimize production cost uncertainty.

**FIGURE 9:**
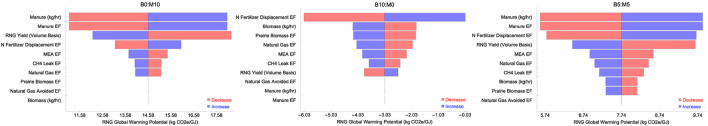
Sensitivity analysis of Renewable Natural Gas (RNG) Global Warming Potential (GWP) to ±20 changes in RNG yield, and material flow and emission factors.

Figure 9 shows the sensitivity analysis results for the RNG GWP. The key parameters for GWP are the manure and biomass factors, the nitrogen fertilizer displacement, and the RNG yield. Twenty percent changes in these parameters vary the GWP by up to 5 kg CO_2_ eq./GJ There are many reasons why these factors could vary as discussed previously. Therefore, these results highlight the importance of improving our understanding of how these factors vary across different ecological scenarios.

## Discussion

The CH_4_ outputs from the ADM1 model for both mono-digestion scenarios fit into the reviewed literature. Reviewed CH_4_ yields from the mono-digestion of cattle manure ranged from 181 mL/gVS to 270 mL/gVS (Kafle and Chen, 2016; Liu et al., 2017; Moset et al., 2017; Tsapekos et al., 2018; Varol and Ugurlu, 2017). The CH_4_ yields for the mono-digestion of switchgrass have a larger range within the literature, between 104 and 309 mL/gVS (Frigon et al., 2012; Labatut et al., 2011; Massé et al., 2010; Zheng et al., 2015). Massé et al. (2010) theorized this trend is not due to the energy content of the biomass changing, but instead due to the digestible material becoming more difficult to access from an increase in non-digestible material.

During a review of various feedstocks under co-digestion, Ma et al. (2020) determined that the mean of CH_4_ produced via co-digested cattle manure increased by 38%. Zheng et al. (2015) and Awais et al. (2016) reported a CH_4_ increase from the co-digestion of manure and switchgrass without pretreatment ranged from 25% to 39%. As our largest CH_4_ increase from co-digestion was less than 6%, our model could be enhanced to capture this synergistic relationship more accurately. This relationship may be better represented if feedstock characterization and calibration was based on experimentation rather than literature. Additionally, integration of gas curves for estimation of the degradation coefficient (d) may represent the co-digestion scenarios more accurately, instead of calculating d. Our biogas prediction model also had a large amount of CO_2_ present within the biogas, as the CH_4_ content of the biogas is typically around 55%–80% (Lusk, 1998).

The estimated MFSPs are largely dependent on the gas produced as there is an inverse relationship seen between the gas production rates and MFSPs. The estimated MFSP of co-digestion scenarios may be reduced further if the synergistic relationship was entirely captured within our model. The difference in feedstock price does not have a large impact on the MFSP as more manure is required to reach the VS digester feed. As biochar production increases with larger amounts of biomass utilized, the MFSP further decreases. If the biochar costs were set higher, this offset would be more significant and could favor scenarios with a larger biomass ratio.

Hegde and Trabold (2019) reported integration of the co-digestion of animal manure not only increases the economic efficiency of a commercial-scale AD but also increases system stability. Efficient use of feedstocks and co-digestion implementation can decrease the MFSP of RNG. While the biogas production requires higher capital costs, the additional sales revenue from an increase in RNG produced leads to a lower MFSP. Additionally, with our high CO_2_ makeup in the biogas from the ADM1 model, our estimation of downstream capital costs may be larger than they would be in reality.

The environmental assessments show a large difference for all impact categories analyzed based on the feedstock used. This is largely due to the manure being an avoided waste, as avoided manure decreases the amount of CH_4_, nitrous oxide (N_2_O), and ammonia emitted to the atmosphere (Havukainen et al., 2020; Sajeev et al., 2018). The impacts seen from biomass cultivation can be attributed to the use of resources such as diesel and fertilizers, which can result in ecosystem eutrophication, ozone depletion, and acidification (Larson, 2006; Zah et al., 2007). Additionally, the use of diesel for farm applications can also result in non-carcinogenics, ecotoxicity, and acidification impacts (Ashworth et al., 2015).

Cultivation of perennial biomass crops can have positive effects on the land, apparent by the beneficial impacts within the ecotoxicity category. Perennials established on marginal land can lead to soil improvements such as a decrease in soil erosion and an increase in the amount of atmospheric CO_2_ captured (Fernando et al., 2018; Guzman et al., 2014; Voigt et al., 2012). Not all biomass requires the same amount of system inputs, as the cultivation of prairie biomass can require lower levels of fertilizer when compared to traditional crops, due partially to efficient absorption within the root system (Cadoux et al., 2012; Hill, 2009). For example, switchgrass was reported to require less diesel fuel, pesticides, herbicides, and phosphorus nutrients than traditional row crops (Ashworth et al., 2015).

Allocation methods are typically based on the physical properties of the products themselves, such as energy or mass. Physical allocation is considered to be the most scientifically sound method of allocation methodology, as it is based on measurable properties of each product, capturing the relationships of the system (Timonen et al., 2019). Economic allocation, dependent on the product’s price, has received criticism for being an unreliable method for impact assignment as it is dependent on the product’s market and human preferences (Wilson et al., 2021). Allocation methods also typically do not consider the use of the product, such as the nutrient capacity of biochar.

Each scenario’s estimated global warming potential falls far below the global warming potential for diesel, estimated at 86 kg CO_2_ eq./GJ (Department for Transport, 2008). The utilization of RNG as a transportation fuel avoids the production and combustion of traditional diesel on roads (Ardolino et al., 2018). Additionally, RNG as a transportation fuel can improve the local air quality by reducing the particulate matter and N_2_O emitted when compared to traditional fuel (U.S. EPA, 2024). Patterson et al. (2013) reported the optimization of an RNG system can further reduce CO_2_ emissions. This relationship may further be seen in our co-digestion LCA results if the ADM1 had further captured the synergistic relationship between cattle manure and prairie biomass.

These results highlight opportunities to leverage co-digestion for reducing costs and environmental impacts of RNG production. Additional strategies that could benefit RNG production include novel reactor designs, electrochemical upgrading technologies, and advanced biomass pretreatment technologies (Uddin and Wright 2023). Future assessments are needed to develop an understanding of the potential for these emerging technologies.

## Conclusion

The addition of co-digestion within a system can create better nutrient levels, therefore increasing AD efficiency and higher gas production levels. As seen with B1:M9, this efficiency leads to smaller MFSPs. As our model did not accurately capture the synergistic relationship between manure and prairie biomass, we expected to see this relationship for more co-digestion scenarios at a larger level. Further analysis should be completed on our ADM1 model implementation with additional integration from laboratory analysis. This may help to estimate TEA and LCA parameters more accurately for the co-digestion scenarios. This could also provide additional ADM1 validation for future models adopting ADM1 for similar economic and environmental analysis for real-world applications. Furthermore, additional factors, like reactor configuration, pretreatment methods, and catalysts could be considered to expand ADM1’s capabilities.

While biomass cultivated for co-digestion can increase environmental and health burdens due to the use of resources, prairie biomass can require fewer materials for cultivation than traditional crops. These feedstocks could make an ideal energy crop for co-digestion if the inclusion would increase the efficiency and CH_4_ production of the system. Establishing prairie biomass can improve ecotoxicity levels by positively influencing the local environment, such as reducing soil erosion. RNG produced from the co-digestion of manure and prairie biomass can increase CH_4_ levels, avoid environmental and health impacts from manure, and further improve ecosystem health from prairie biomass cultivation.

## Data Availability

The original contributions presented in the study are included in the article/Supplementary Material, further inquiries can be directed to the corresponding author.
