# Supplementary material for: Advancing the predictive techno-economic and lifecycle assessment of prairie grass and manure co-digestion for renewable natural gas applications
Source: Front Bioeng Biotechnol. 2025 Sep 30;13:1651510. doi: 10.3389/fbioe.2025.1651510 (PMC12518338; doi:10.3389/fbioe.2025.1651510)
Supplement: Supplementary file 1 [file Table1.docx]

## Supporting Information.

Table SI 1. ADM1 model calibration parameters.

| **Scenario** | **Parameter variable** | **Mono-digestion of biomass** | **Co-digestion of biomass and manure** | **Mono-digestion of manure** |
| --- | --- | --- | --- | --- |
| Hydrolysis rate of carbohydrates | k_hyd,ch_ | 0.6865 | 10 | 0.31 |
| Hydrolysis rate of proteins | k_hyd,pr_ | 0.2446 | 10 | 0.31 |
| Hydrolysis rate of lipids | k_hyd,li_ | 0.1216 | 10 | 0.31 |
| Maximum uptake rate for acetate utilizers | k_m,ac_ | 7.1 | 7.64 | 3.6 |
| Maximum uptake rate for propionate | k_m,pro_ | 5.5 | 8.5 | 13 |
| Maximum uptake rate for butyrate | k_m,c4_ | 13.7 | 20 | 20 |
| Disintegration constant | k_m,dis_ | 0.26 | 0.45 | 0.05 |
| Source |  | Biernacki et al., 2013; Thamsiriroj & Murphy, 2011; Wichern et al., 2009 | Bułkowska, et al., 2015; Shi et al., 2014 | Lübken et al., 2007; Wichern et al., 2007 |

Table SI 2. Calculated parameters and results from ADM1.

| **Parameter variable** | **Prairie Biomass** | **Cattle Manure** | **Units** |
| --- | --- | --- | --- |
| X_c_ | 1136.1 | 207.1 | kgCOD/m^3^ |
| NfE | 36.149 | 41.149 | % TS |
| f_Ch_xc_ | 0.21005 | 0.29439 | % |
| f_I_xc_ | 0.63900 | 0.50500 | % |
| f_P_xc_ | 0.07772 | 0.16214 | % |
| f_L_xc_ | 0.07323 | 0.03847 | % |
| S_su_ | 0 | 22.36441 | kgCOD/m^3^ |
| S_aa_ | 0 | 5.59110 | kgCOD/m^3^ |
| S_fa_ | 0 | 1.65662 | kgCOD/m^3^ |
| S_I_ | 0 | 11.80344 | kgCOD/m^3^ |
| d | 0.153439 | 0.15073 | % |

~~
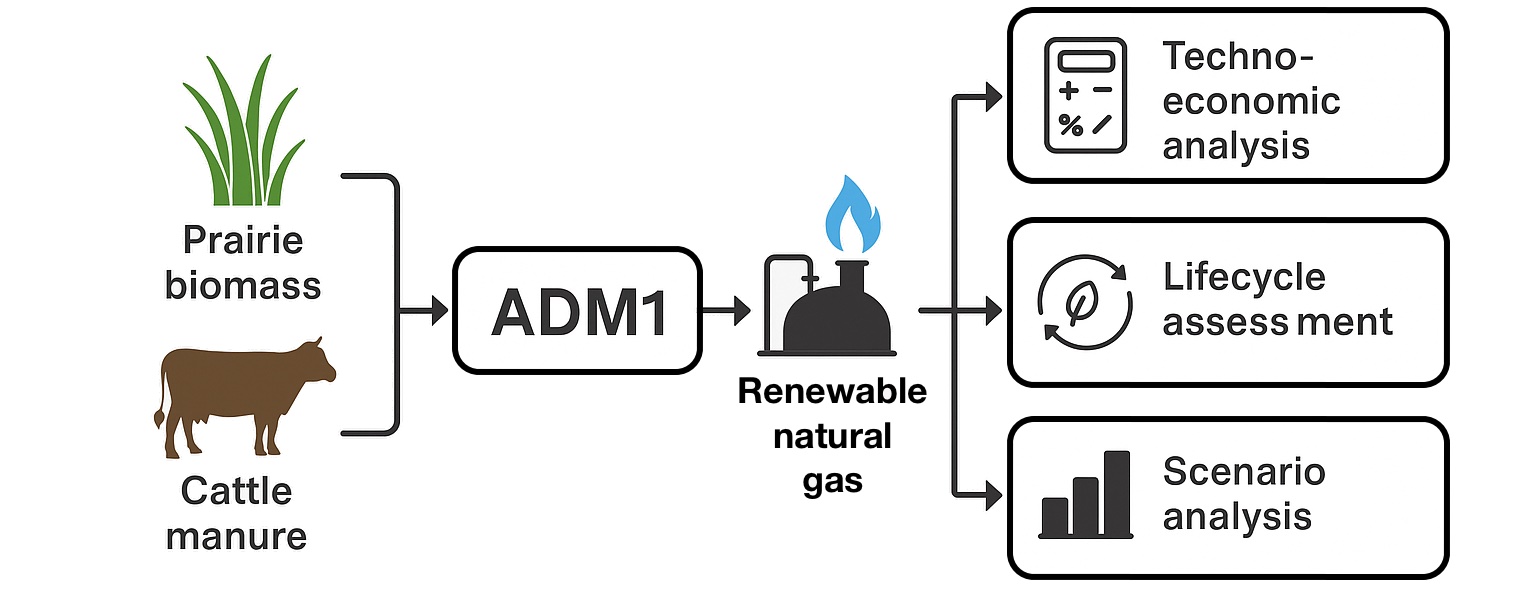
~~
